# Supplementary material for: Adherence to self-care practices and associated factors among heart failure patients in Ethiopia: A systematic review and meta-analysis
Source: PLoS One. 2023 Aug 23;18(8):e0288824. doi: 10.1371/journal.pone.0288824 (PMC10446213; doi:10.1371/journal.pone.0288824)
Supplement: Attachment — Submitted filename: Reviewer comment.docx [file pone.0288824.s004.docx]

Independent Review Report

**Evaluation**

The evaluation is for the review article titled “Self-care Practices and associated factors among heart failure patients in Ethiopia: A systematic review and Meta-Analysis”

With the present study, the following areas are observed, commented and recommended.

1. Throughout the main text, it was sequential and well organized.
2. It requires grammatical corrections throughout the document.
3. This manuscript has a sum up of novelty.

**Comments to the author/s**

**Abstract:**

1. Try to avoid abbreviation of words in the abstract part
2. “We have used three databases such as Pub Med, Science Direct and Google Scholar.

Why do you exclude other important databases that you might get potential primary studies from?

3. Heterogeneity was evaluated using the I2 tests and the Cochrane Q test statistic.

What is the advantage of performing the Q test in addition to the I2 test to assess heterogeneity?

4. To examine publication bias, a funnel plot, Egger's weighted regression, and Begg's test were utilized. What is the importance of using different tests?
5. “The extent of self-care behaviour adherence is shown to be low among heart failure patients”. What is the standard reference to say “low or high”?

**Method**

6. Please use the uniform reference citation system
7. Please try to strictly adhere to all the PRISMA 2020 guidelines to write up your paper, especially the method section.
8. Does this review registered on PROSPERO?
9. What is your last search date included in the method part?
10. What you have done if a study has low quality or higher risk?
11. “Finfinnee" is not a legal name, not yet known
12. What you have done if there is heterogeneity and publication bias between the primary studies?
13. How to perform the pooled effect of odds ratio to identify the factors associated with good selfie care? Please show on your paper as it is critical.

**Result**

14. Table 1 column 6 gender (male). What is this???
15. Why you have performed sub-group analysis and sensitivity test?
